# Integrative proteomic and lipidomic analysis of Kaili Sour Soup-mediated attenuation of high-fat diet-induced nonalcoholic fatty liver disease in a rat model

**DOI:** 10.1186/s12986-021-00553-4

**Published:** 2021-03-10

**Authors:** Shuo Cong, Zhengchao Li, Lei Yu, Yongmei Liu, Yaxin Hu, Ying Bi, Mingliang Cheng

**Affiliations:** 1 School of Basic Medicine Sciences, Guizhou Medical University, Guiyang City, China; 2 Guizhou Cancer Hospital, Guiyang City, China; 3 Graduate School of Guizhou Medical University, Guiyang City, China; 4 Maternal and Child Health Hospital of Guiyang City, Guiyang City, China; 5 Department of Infectious Diseases, Affiliated Hospital of Guizhou Medical University, 28 Guiyi Street, Guiyang City, 550004 Guizhou Province China

**Keywords:** Kaili Sour Soup, Nonalcoholic fatty liver disease, Proteomics, Lipidomics

## Abstract

**Background:**

Nonalcoholic fatty liver disease (NAFLD) is the most prevalent liver disease and is characterized by excessive fat accumulation. Kaili Sour Soup, a food typical of Guizhou Province, is believed to have significant health benefits. Thus, we aimed to identify and assess the impact of Kaili Sour Soup on NAFLD and its underlying mechanism using integrative proteomic and lipidomic analysis.

**Methods:**

A high-fat diet and male Wistar rats were used to construct a NAFLD rat model. Haematoxylin and eosin (HE) and Oil Red O staining analyses were used to perform the histologic examination. Proteomic analysis was utilized to systematically identify the global protein profile in NAFLD with and without Kaili Sour Soup treatment. Western blot assays were used to verify the expression of proteins screened by proteomic analysis. Lipidomic analysis was performed to screen lipid metabolism in NAFLD with and without Kaili Sour Soup treatment.

**Results:**

Kaili Sour Soup alleviated high-fat diet (HFD)-induced fatty liver and had a normalizing effect on physiological and biochemical indicators of NAFLD, including body weight, liver weight, liver index, total cholesterol (TC), triglyceride (TG), alanine aminotransferase (ALT), aspartate aminotransferase (AST) and insulin resistance level of homeostasis model assessment (HOMA-IR). Kaili Sour Soup decreased the levels of 13 proteins (Tmem44, Rnaseh2b, Gstm6l, LOC100910877, Rufy4, Slc12a2, Pcif1, P4503A1, Sult1e1, Nop53, AABR07065656.4, AABR07065789.3) that were upregulated by HFD and increased the levels of 3 proteins (Sult1c2, Sult1c2a, Snrnp48) that were downregulated by HFD. Kaili Sour Soup attenuated the HFD-induced increase in acyl carnitine (AcCa) and enhanced the HFD-induced decreases in gangliosides (GM3) and lysophosphatidylserine (LPS) in the NAFLD rat model.

**Conclusions:**

Altogether, this study revealed that Kaili Sour Soup attenuated HFD-induced fatty liver and systematically identified abnormal proteins and lipids involved in the role of Kaili Sour Soup in a NAFLD rat model.

**Supplementary Information:**

The online version contains supplementary material available at 10.1186/s12986-021-00553-4.

## Introduction

Kaili Sour Soup, a food typical of Guizhou Province, is naturally fermented using traditional techniques, with red peppers and tomatoes as the main ingredients. While a traditional food, people are confident that Kaili Sour Soup is not just a seasoning but also has great health benefits. Enzymes, probiotics, prebiotics, lactic acid, acetic acid, calcium, phosphorus, iron and other beneficial ingredients in sour soup [1] have significant human health benefits. Thus, these factors are worthy of in-depth exploration and research, especially given the role that they play in the body's metabolic processes.

As the name suggests, nonalcoholic fatty liver disease (NAFLD), defined as fatty infiltration of the liver in the absence of alcohol abuse, is one of the most prevalent chronic liver disorders in China and Western countries [1]. NAFLD is classically subdivided into nonalcoholic fatty liver (NAFL), which is characterized by fatty infiltration without hepatocellular injury, and nonalcoholic steatohepatitis (NASH), which is characterized by inflammation and ballooning, with or without fibrosis [2]. A high-fat and refined carbohydrate diet is the major inducer of hyperinsulinaemia and fatty liver. To this point, dietary intervention has been the most important clinical intervention for NAFLD and NASH, but dietary intervention cannot treat NAFLD and NASH [3]. Recently, a survey in our lab revealed that fat metabolism is superior in members of the local population who prefer Kaili Sour Soup compared to the non-Kaili Sour Soup preference group (data not shown). However, the mechanism underlying this phenomenon is largely unknown. Furthermore, the impact of Kaili Sour Soup on NAFLD and its underlying mechanism remain largely unexplored to date.

Technological advances in proteomics and lipidomics allow for the systematic measurement of overall protein and lipid metabolism during the physiological and pathological processes of various diseases [4, 5]. Analysis of the changes and balance in the overall proteome and lipidome helps us better understand the molecular mechanisms underlying the development and progression of NAFLD. Several studies have focused on overall protein and lipid metabolism in NAFLD.

Thus, this study aimed to investigate the impact of Kaili Sour Soup on NAFLD and further dissect its underlying molecular mechanisms by systematically screening overall protein and lipid metabolism in NAFLD after Kaili Sour Soup treatment. Ultimately, this study first induced NAFLD in a rat model via a high-fat diet and treated them with Kaili Sour Soup. Next, we employed the TMT Proteome Test to analyse alterations in the proteome and applied liquid chromatography coupled to mass spectrometry (LC–MS) to analyse changes in lipid metabolism in the NAFLD rat model with or without treatment with Kaili Sour Soup.

## Methods and materials

### Animal care and experimental study

All of the animal experiments presented in this research were approved and supervised by the Animal Ethics Committee of Guizhou Medical University, and all operations were performed in accordance with the Guide for the Care and Use of Laboratory Animals of China.

Kaili Sour Soup was purchased from Guizhou Lianghuanzhai Catering and Entertainment Management Co., Ltd. (Kaili, Guizhou, China). Thirty-seven male Wistar rats (6 weeks old) were purchased from Liaoning Changsheng Biotechnology Co., Ltd. (Benxi City, Liaoning, China) and fed a normal standard diet for approximately 1 week. The rats were then randomly divided into four groups: (i) ND group: normal standard feed + purified water gavage for 12 weeks (n = 8); (ii) NDS group: normal standard feed + Kaili Sour Soup gavage for 12 weeks (n = 8); (iii) HFD group: high-fat feed + purified water gavage for 12 weeks (n = 9); and (iv) HFS group: high fat feed + Kaili Sour Soup gavage for 12 weeks (n = 12). The normal standard diet was composed of 80% carbohydrates, 15% protein and 5% fat. The high-fat diet protein:carbohydrate:fat ratio provided an energy ratio of 20%:20%:60% and had the following composition: casein ≥ 88%: 256.4 g/kg, L-cystine: 3.8 g/kg, maltodextrin: 147.3 g/kg, sucrose: 101.3 g/kg, cellulose: 64.1 g/kg, soybean oil: 32.1 g/kg, lard: 314.1 g/kg, mineral AIN-93: 44.9 g/kg, vitamin AIN-93: 12.8 g/kg, choline chloride: 3.2 g/kg, and cholesterol: 20 g/kg. High-fat feed was prepared according to this formula by the Guangdong Medical Laboratory Animal Centre (Foshan City, Guangdong, China). Kaili Sour Soup and purified water were given at 1 mL/100 g body weight/day at 12 o'clock every day, 6 days a week. All animals were housed under a 12-h diffuse light/12-h dark cycle at 23 ± 2 °C and 60 ± 10% relative humidity and were provided sufficient food and water.

### Histologic examination

Rats were euthanized by an intraperitoneal injection of 10% chloral hydrate. The same part of each liver was harvested and fixed in 4% paraformaldehyde (PFA) immediately. The tissues were then sent to the Department of Pathology, Guizhou Medical University (Guiyang City, Guizhou, China), for haematoxylin and eosin (HE) and Oil Red O staining. The results were observed and recorded using a light microscope (Olympus, Tokyo, Japan).

### Proteomic analysis using Tandem Mass Tag-LC–MS/MS

Proteomic analysis using tandem mass tag-LC–MS/MS was performed by Applied Protein Technology (Shanghai, China). For protein extraction and peptide digestion, proteins from rat liver tissue samples were extracted using the SDT (4% (w/v) SDS, 100 mM Tris/HCl pH 7.6, 0.1 M DTT) lysis method, and the concentrations were measured using a BCA detection kit (Junxin Biotech, Suzhou, China). Equal amounts of protein were used for trypsin digestion using the filter aided sample preparation (FASP) method as described previously [6]. The concentration and purity of the peptides were measured using a microspectrophotometer (Thermo Fisher Scientific, MA, USA). Equal amounts of peptides (100 μg) were used for TMT labelling with a TMT labelling kit, TMT Mass Tagging Kits and Reagents (Thermo) according to the manufacturer’s instructions. For High pH Reversed-Phase Peptide Fractionation, the labelled peptides in each group were mixed in equal amounts and graded using the High pH Reversed-Phase Peptide Fractionation Kit. First, acetonitrile and 0.1% trifluoroacetic acid (TFA) were used for column equilibration. Then, the mixed labelled peptide samples were loaded. Pure water was added and centrifuged at a low speed for desalting treatment. Finally, high pH acetonitrile solutions of increasing concentrations were used to perform gradient elution of the column-bound peptide. Each eluted peptide sample was vacuum dried and reconstituted with 12 μL 0.1% FA of the freeze-dried sample, and the peptide concentration was determined by an OD280 measurement. For LC–MS/MS data acquisition, each graded sample was separated using the HPLC liquid phase system Easy-nLC with a nanolitre flow rate. Briefly, the loading column (Thermo Scientific Acclaim PepMap 100, 100 μm * 2 cm, nanoViper C18) was equilibrated with 95% buffer (0.1% formic acid in water). Then, the sample was loaded and passed through an analytical column (Thermo Scientific EASY column, 10 cm, ID75 μm, 3 μm, C18-A2) for separation, with a flow rate of 300 nL/min. Next, the sample was analysed using a Q Exactive mass spectrometer using the positive ion method. The scanning range of the precursor ion was 300–1800 m/z. The resolution of primary mass spectrometry was 70,000 at 200 m/z. The automatic gain control (AGC) target was 1e6. The maximum IT was 50 ms, and the dynamic exclusion time (dynamic exclusion) was 60.0 s. The mass-to-charge ratios of peptides and peptide fragments were collected according to the following method: 20 fragment maps (MS2 scan) were collected after each full scan, the MS2 activation type was HCD, the isolation window was 2 m/z, and the secondary mass spectrometry resolution rate was 17,500 at 200 m/z (TMT 6-plex) or 35,000 at 200 m/z (TMT 10-plex). Protein identification and quantitative analysis were performed using Mascot 2.2 and Proteome Discoverer 1.4, respectively. For bioinformatics analysis, Blast2GO was used to perform GO annotation of the target protein collection. KAAS (KEGG Automatic Annotation Server) software was employed to annotate the target protein collection using KEGG pathway analysis. Fisher's exact test was used for enrichment analysis of the GO annotation, and the KEGG annotation ComplexHeatmap R package (R Version 3.4) was applied for protein cluster analysis. The STRING (http://string-db.org/) database and CytoScape software (version number: 3.2.1) were used for protein interaction network analysis.

### Western blots analysis

Radioimmunoprecipitation assay (RIPA) lysis buffer (Junxin Biotech, Suzhou, China) was employed to extract the protein. A bicinchoninic acid (BCA) protein assay (Junxin Biotech, Suzhou, China) was used to detect the concentration of protein. Protein (50 μg) was loaded and separated by SDS–polyacrylamide gel electrophoresis, transferred onto polyvinylidene fluoride (PVDF) membranes (Millipore, Billerica, USA), blocked in bovine serum albumin (BSA) (Millipore, Billerica, USA), probed with primary antibodies and then incubated with the corresponding secondary antibodies. The results were visualized by electrochemiluminescence (Junxin Biotech, Suzhou, China). The antibodies were as follows: anti-Rnaseh2b (1:1000 dilution; Proteintech, USA), anti-Slc12a2 (1:1000 dilution; Affinity Biosciences, China), anti-Pcif1 (1:1000 dilution; Cell Signaling Technology, USA), anti-P4503A1 (1:1000 dilution; Abcam, USA), anti-Sult1e1 (1:1000 dilution; Abcam, USA), anti-Sult1c2 (1:1000 dilution; Proteintech, USA), anti-Snrnp48 (1:1000 dilution; Abcam, USA), and anti-β-Actin (1:4000 dilution; Abcam, USA).

### Lipidomic analysis using LC–MS

Lipidomic analysis using LC–MS was performed by Applied Protein Technology (Shanghai, China). For sample preprocessing, after slowly thawing at 4 °C, the samples (30 mg) were placed in 2 mL MP tubes. Then, 200 μL of water, 240 μL of precooled methanol and 800 μL of MTBE were added one by one. After vortexing, the mixtures were sonicated for 20 min in a low-temperature water bath. After incubation at room temperature for 30 min, the samples were centrifuged at 14,000*g* at 10 °C for 15 min. The upper organic phase was collected and dried with nitrogen, and then 200 µL of 90% isopropanol/acetonitrile solution was added. The samples were then vortexed and centrifuged at 14,000*g* at 10 °C for 15 min. Finally, the supernatant was collected for LC–MS analysis.

For LC–MS analysis, the samples were separated using a UHPLC Nexera LC-30A ultra-high-performance liquid chromatography system and analysed with a Q Exactive Plus mass spectrometer (Thermo Scientific™) using electrospray ionization (ESI) positive and negative ion modes. The mass-to-charge ratio of lipid molecules to lipid fragments was collected according to the following method: 10 fragment maps (MS2 scan, HCD) were collected after each full scan. MS1 has a resolution of 70,000 at M/Z 200, and MS2 has a resolution of 17,500 at M/Z 200.

### Data analysis

LipidSearch software version 4.1 Thermo Scientific™ was used for peak identification, lipid identification (secondary identification), peak extraction, peak alignment and quantification. The main parameters were precursor tolerance: 5 ppm, product tolerance: 5 ppm, and product ion threshold: 5%. For the data extracted by LipidSearch, the lipid molecules in the group with 50% missing values were deleted, and the total peak area of the data was normalized. The application software SIMCA P 14.1 (Umetrics, Umea, Sweden) was used for pattern recognition. After the data were preprocessed using Pareto scaling, multidimensional statistical analysis was performed, including unsupervised principal component analysis (PCA), supervised row least squares discriminant analysis (PLS-DA) and orthogonal partial least squares discriminant analysis (OPLS-DA). Single-dimensional statistical analysis included Student’s t-test and mutation multiple analysis. R software was used to draw volcano maps and perform hierarchical cluster and correlation analyses. For comparisons between the HFD and ND groups, *p ≤ 0.05 and **p ≤ 0.01 represent statistical significance, and ^∆^*p* ≤ 0.05 and ^∆∆^*p* ≤ 0.01 represent statistical significance for comparisons between the HFD and HFDS groups.

## Results

### Kaili Sour Soup improves multiple markers in the NAFLD rat model

**Fig. 1:**
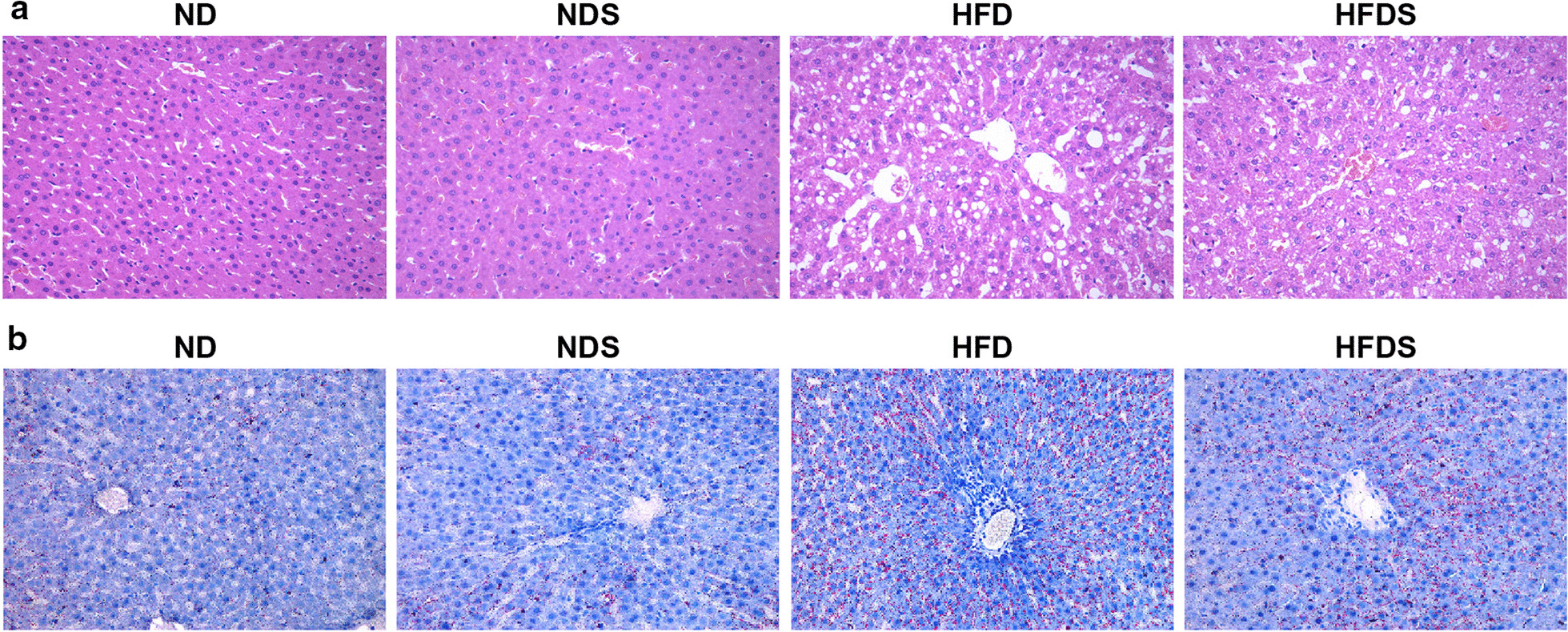
Kaili Sour Soup attenuates fat formation in the liver in the NAFLD rat model. **a** Representative images of HE staining of rat livers. **b** Representative images of Oil Red O staining of rat livers. The rats were divided into four groups: normal diet group (ND), normal diet + Kaili Sour Soup group (NDS), high-fat diet group (HFD) and high-fat diet + Kaili Sour Soup group (HFDS). The HE assay was utilized to measure fat formation in the rat livers. N = 9

To determine whether Kaili Sour Soup impacts NAFLD, we first established an NAFLD rat model by feeding rats a high-fat diet with or without Kaili Sour Soup. Thus, the rats were divided into four groups: normal diet group (ND), normal diet + Kaili Sour Soup group (NDS), high-fat diet group (HFD) and high-fat diet + Kaili Sour Soup group (HFDS). First, the results of HE and Oil Red O staining suggested an increase in fat formation in the livers of the high-fat diet group (Fig. 1a, b). Furthermore, there was an increase in body weight, liver weight, liver index, Lee’s index, total cholesterol (TC), triglyceride (TG), alanine aminotransferase (ALT), aspartate aminotransferase (AST), fasting blood glucose (FBG) level, insulin level (FINS), and insulin resistance level (HOMA-IR) (Table 1). These observations indicated that we successfully established the NAFLD rat model.

**Table 1:** Physiological and biochemical markers in the NAFLD rat model

Index	ND group	NDS group	HFD group	HFDS group
Body weight (g)	461.67 ± 23.89	462.67 ± 38.99	570.83 ± 23.02**	500.50 ± 23.72^∆∆^
Liver weight (g)	14.81 ± 1.05	14.05 ± 1.98	21.01 ± 1.41**	19.24 ± 0.93^∆∆^
Liver index (%)	3.21 ± 0.19	3.03 ± 0.32	3.68 ± 0.23**	3.85 ± 0.12^∆∆^
Lee’s index	3.00 ± 0.08	3.05 ± 0.06	3.19 ± 0.05**	3.10 ± 0.10
TC (mmol/L)	1.43 ± 0.40	1.38 ± 0.30	2.59 ± 0.16**	2.19 ± 0.29^∆∆^
TG (mmol/L)	0.64 ± 0.19	0.67 ± 0.39	1.47 ± 0.26**	1.09 ± 0.16^∆∆^
ALT (U/L)	41.83 ± 6.76	44.87 ± 9.26	106.48 ± 18.35**	87.00 ± 8.10^∆^
AST (U/L)	145.23 ± 17.95	182.08 ± 40.23	429.67 ± 56.46**	364.18 ± 40.04^∆∆^
FBG(mmol/L)	8.01 ± 1.21	8.92 ± 2.07	13.36 ± 1.02**	12.22 ± 1.41
FINS (mIU/L)	22.27 ± 2.17	23.58 ± 1.05	31.80 ± 4.27**	29.31 ± 1.79
HOMA-IR	9.23 ± 1.44	11.25 ± 1.98	15.88 ± 2.12**	13.67 ± 0.53^∆^
FINS (mIU/L)	10.55 ± 0.67	10.73 ± 1.62	18.82 ± 1.20**	16.03 ± 5.54^∆^
HOMA-IR	3.78 ± 0.80	4.37 ± 1.75	11.17 ± 1.12**	8.80 ± 2.07^∆^

Liver index = (liver weight/body weight) × 100%; Lee’s index = (weight × 1000)^(1/3)/length (cm); Total cholesterol (TC); Triglyceride TG; Alanine aminotransferase ALT; Aspartate aminotransferase AST; Fasting blood glucose FBG; Insulin level FINS; Insulin resistance level HOMA-IR = (FINS*FBG)/22.5. *p ≤ 0.05 and **p ≤ 0.01 represented statistically significance between HFD group and ND group; ^∆^*p* ≤ 0.05 and ^∆∆^*p* ≤ 0.01 represented statistically significance between HFDS group and HFD group. n = 6

Next, we aimed to measure the impact of Kaili Sour Soup on the NAFLD rat model. First, the results of HE and Oil Red O staining revealed that Sour Soup reduced fat formation in the livers of the high-fat diet group (Fig. 1a, b). Furthermore, the results revealed that Kaili Sour Soup exerted an anti-NAFLD effect in the NAFLD rat model. Kaili Sour Soup inhibited the increases in body weight, liver weight, liver index, TC, TG, ALT, AST, and HOMA-IR (Table 1). These results showed a protective impact of Kaili Sour Soup in the NAFLD rat model via the attenuation of high-fat diet-induced fatty liver abnormal markers in NAFLD.

Taken together, these observations suggested that Kaili Sour Soup exerted an anti-NAFLD effect in the NAFLD rat model.

### Global proteome response to a high-fat diet treated with Kaili Sour Soup

To examine the global proteome response to Kaili Sour Soup in NAFLD, TMT quantitative proteomics technology was used to systematically analyse the global proteome alterations between the ND and HFD groups as well as between the HFD and HFDS groups. We identified a total of 6158 proteins (Additional file 1: Fig. S1).

**Fig. 2:**
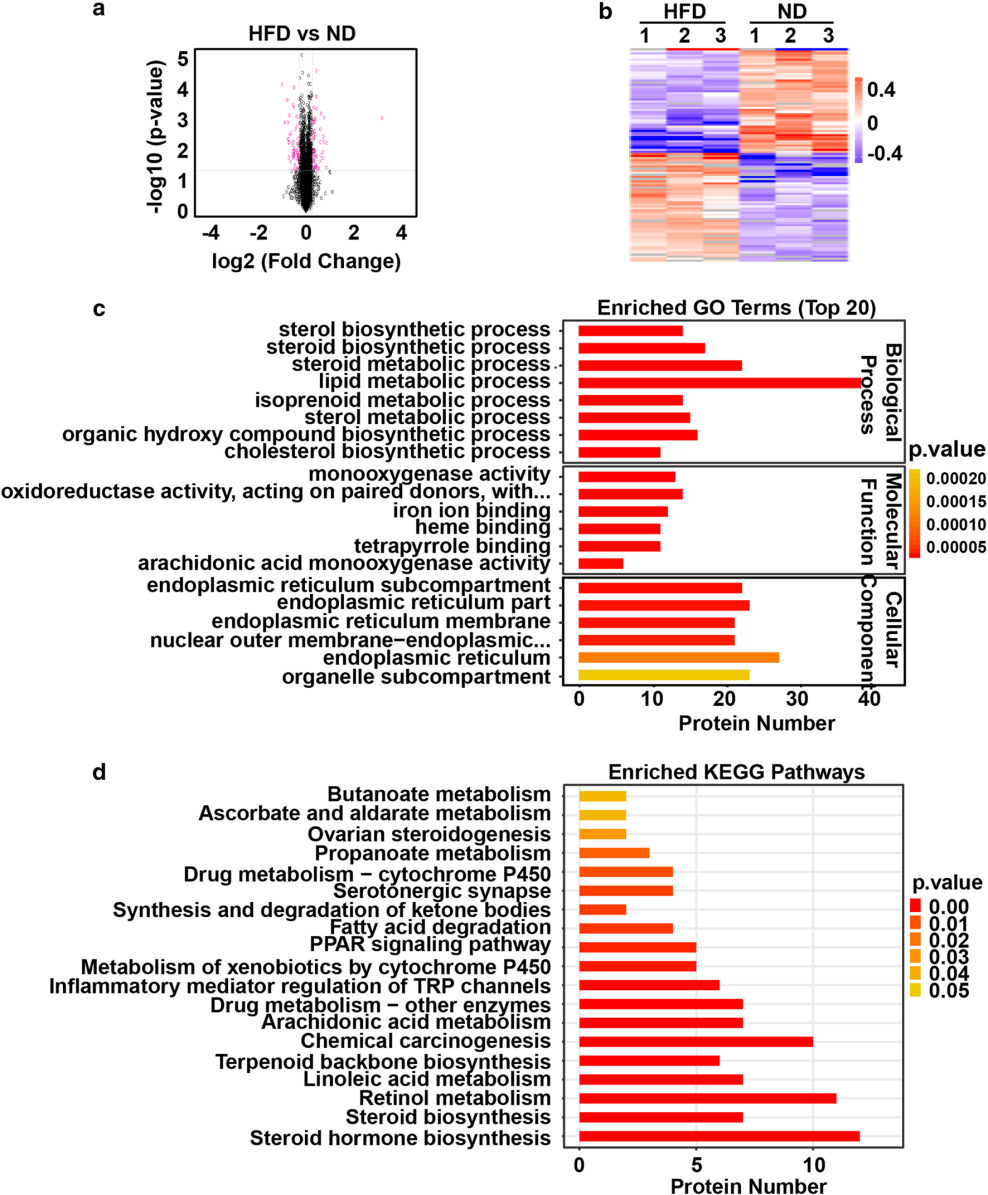
Global proteome response in high-fat diet-treated rats. **a** Volcano map for the HFD_*vs*_ND group. The red dots in the figure represent proteins that are significantly differentially expressed (fold change greater than 1.2 and *p* value < 0.05), and the black dots represent proteins that showed no difference. **b** Hierarchical clustering of proteins in the HFD_*vs*_ND group. Red represents significantly upregulated proteins, blue represents significantly downregulated proteins, and grey represents no quantitative protein information. **c** Gene Ontology (GO) enrichment analysis. **d** Functional enrichment analyses using Kyoto Encyclopaedia of Genes and Genomes (KEGG) pathways

In the HFD_*vs*_ND group, there were 53 upregulated proteins and 49 downregulated proteins (≥ 1.2-fold, *p* ≤ 0.05) (Fig. 2a, b). GO function and KEGG pathway analyses showed that these differentially expressed proteins mainly had functions in binding, catalytic activity, transporter activity, regulating molecular function, and regulating transcriptional regulator activity and were involved in the biological processes of biological regulation and regulation (Fig. 2c, d).

**Fig. 3:**
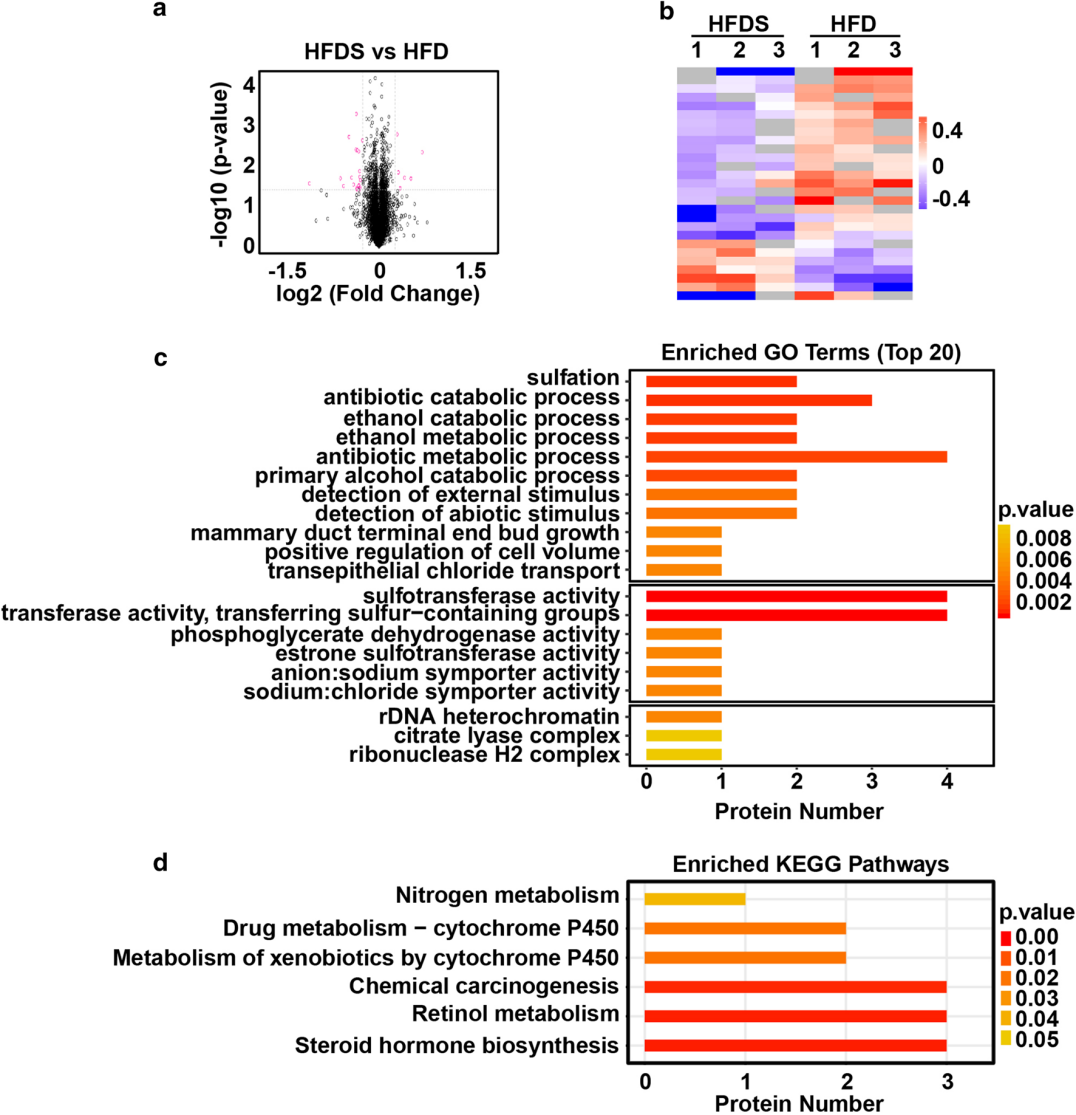
Global proteome response to a high-fat diet treated with Kaili Sour Soup. **a** Volcano map for the HFDS_*vs*_HFD group. The red dots in the figure represent proteins that are significantly differentially expressed (fold change greater than 1.2 and *p* value < 0.05), and the black dots represent proteins that showed no difference. **b** Hierarchical clustering of proteins in the HFDS_*vs*_HFD group. Red represents significantly upregulated proteins, blue represents significantly downregulated proteins, and grey represents no quantitative protein information. **c** Gene Ontology (GO) enrichment analysis. **d** Functional enrichment analyses using Kyoto Encyclopaedia of Genes and Genomes (KEGG) pathways

In the HFDS_*vs*_HFD group, there were 6 upregulated proteins and 21 downregulated proteins (≥ 1.2-fold, *p* ≤ 0.05) (Fig. 3a, b). GO function and KEGG pathway analyses revealed that these differentially expressed proteins mainly functioned in catalytic activity, binding, and transporter activity and were involved in the biological processes of cellular process, metabolic process, biological regulation, regulation of biological process and response to stimulus (Fig. 3c, d).

**Fig. 4:**
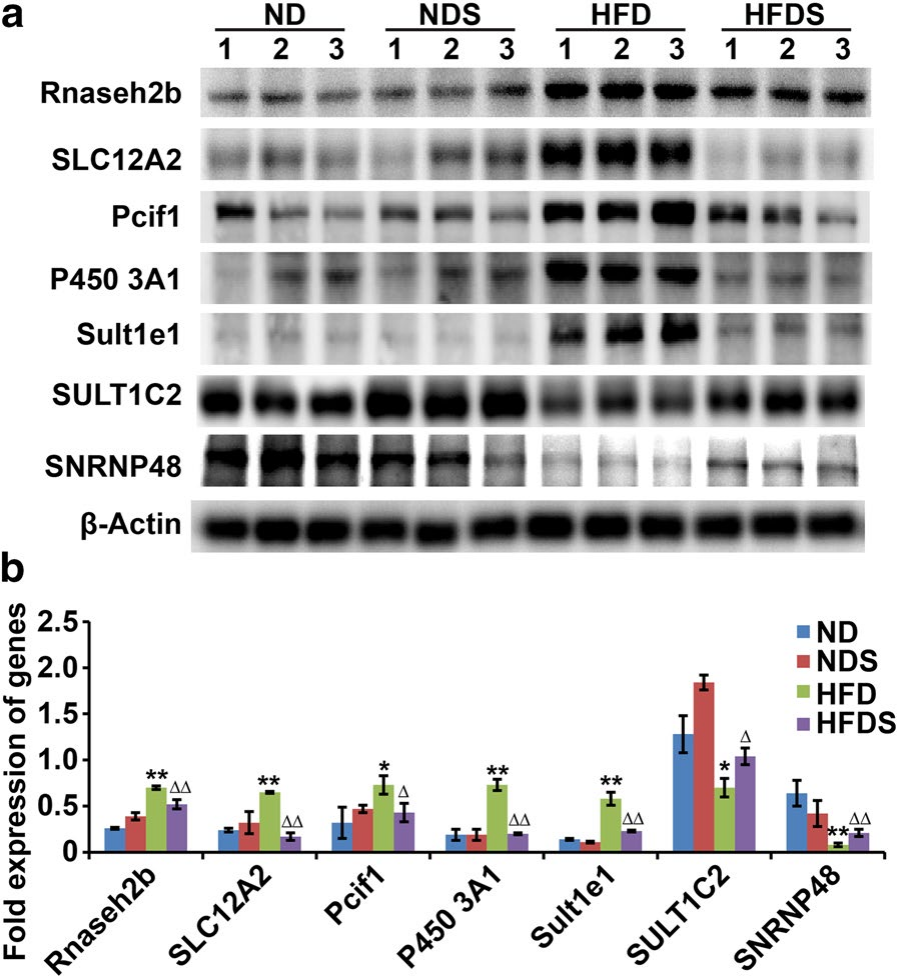
Identification of the changed expression levels of proteins in NAFLD with and without Kaili Sour Soup treatment. **a** and **b** Western blot analysis of the protein expression in NAFLD with and without Kaili Sour Soup treatment. β-Actin served as a reference control

Then, Western blot analysis was used to verify the alteration of protein levels, and the results showed that Rnaseh2b, Slc12a2, Pcif1, P4503A1 and Sult1e1 were upregulated in the HFD group compared to the ND group and downregulated in the HFDS group compared to the HFD group (Fig. 4a). Meanwhile, Sult1c2 and Snrnp48 were downregulated in the HFD group compared to the ND group and upregulated in the HFDS groups compared to the HFD group (Fig. 4b).

In summary, these results revealed that Kaili Sour Soup affected the global proteome response to a high-fat diet.

### Global lipidome response to a high-fat diet treated with Sour Soup

**Fig. 5:**
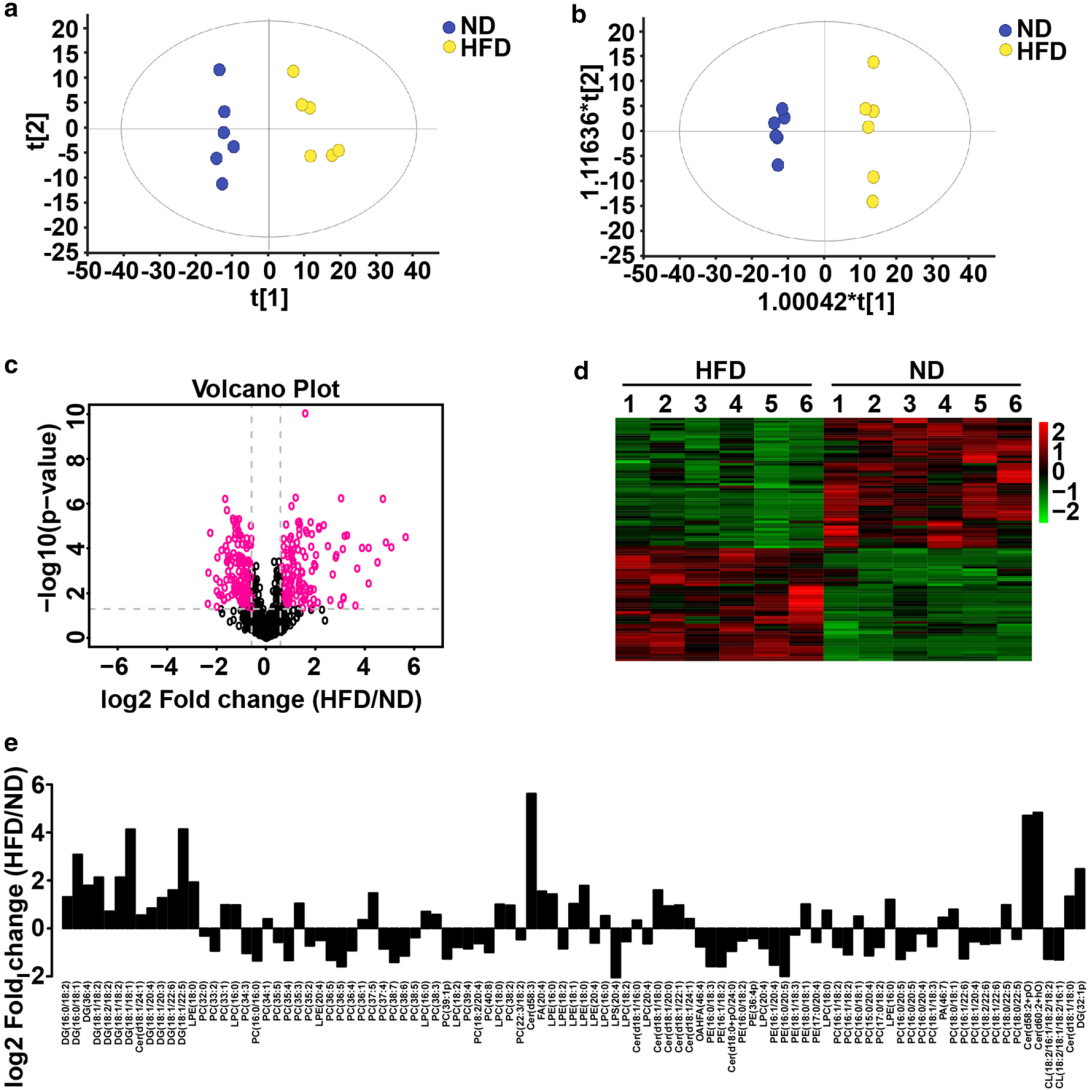
Global lipidome response in high-fat diet-treated rats. **a**, **b** Score plots of PCA and OPLS-DA in the HFD_*vs*_ND group (n = 6). **c** Volcano plot for the HFD_*vs*_ND group. Red dots represent significantly different lipids (FC > 1.5 p value < 0.05). **d** Heatmap visualization of the differentially abundant metabolites. **e** Analysis of the multiples of variation of significantly different lipid molecules in the HFD_*vs*_ND group

**Fig. 6:**
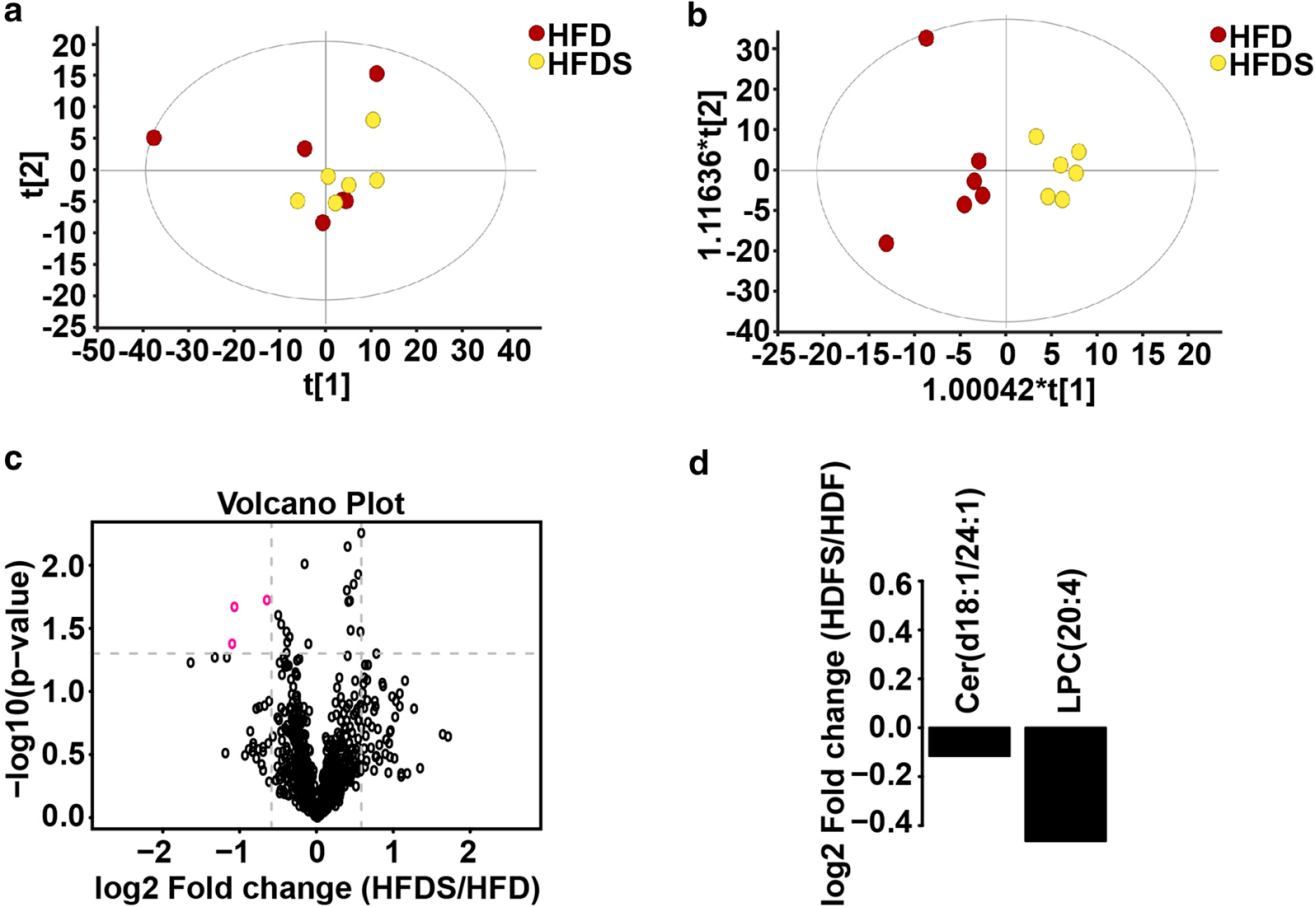
Global lipidome response to a high-fat diet in the Kaili Sour Soup group. **a**, **b** Score plots of PCA and OPLS-DA in the HFDS_*vs*_HFD group (n = 6). **c** Volcano plot for the HFD_*vs*_ND group. Red dots represent significantly different lipids (FC > 1.5 *p* value < 0.05). **d** Analysis of the multiples of variation of significantly different lipid molecules in the HFDS_*vs*_HFD group

The reported important role played by lipids in NAFLD was revealed by the marked changes in proteins functioning in lipid metabolism identified by our global proteome analysis, thus indicating the potential of the extensive alteration of the cell lipidome in NAFLD [7, 8]. Therefore, we hypothesized that Kaili Sour Soup might exert its effects by altering the cell lipidome in NAFLD. To test this hypothesis, we performed LC–MS-based experiments to systematically examine the lipid profiles of NAFLD with or without Kaili Sour Soup treatment. According to the PCA results, the metabolomic profiles were clearly separated between the HFDS, HFD and ND groups (Figs. 5a and 6a). The supervised OPLS-DA model was then utilized to determine the differences between the HFDS, HFD and ND groups, and the results showed that the models were successfully constructed (Figs. 5b and 6b). Taken together, these results displayed dramatic metabolic alterations in rat livers among the HFDS, HFD and ND groups.

The lipidomic analysis results identified a total of 817 lipid species (Additional file 2: Fig. S2) and 23 classes of lipid compounds in the positive and negative ion modes (Additional file 3: Fig. S3). In the HFD_*vs*_ND group, there were 45 upregulated lipids and 52 downregulated lipids (≥ 1.5-fold, *p* ≤ 0.05) (Fig. 5c–e). In the HFDS_*vs*_HFD group, there were no upregulated lipids and 2 downregulated lipids (≥ 1.5-fold, *p* ≤ 0.05) (Fig. 6c, d).

Collectively, these results indicate a large alteration in the global lipidome response to a high-fat diet. Surprisingly, a small change in the global lipidome occurred in the HFDS group compared to the HFD group, indicating that Kaili Sour Soup has a weak impact on the global lipidome in the NAFLD rat model.

## Discussion

This study first revealed the protective effect of Kaili Sour Soup against NAFLD and then systematically investigated the proteome and lipid metabolism in a NAFLD rat model with and without Kaili Sour Soup treatment. This study elucidated the molecular mechanism underlying the protective role played by Kaili Sour Soup in NAFLD and provided a theoretical basis for the prevention and treatment of NAFLD using Kaili Sour Soup.

As a food typical of Guizhou Province, Kaili Sour Soup is thought to exert benefits on fat metabolism. However, few studies have focused on the impact of Kaili Sour Soup on NAFLD and its underlying mechanism. Here, we first established a NAFLD rat model and found that multiple markers of NAFLD were increased in the NAFLD rat model, including body weight, liver weight, liver index, Lee’s index, TC, TG, ALT, AST, FBG, FINS and insulin resistance level. Most of these increased markers in NAFLD were improved by Kaili Sour Soup, including liver weight, liver index, TC, TG, ALT, AST and insulin resistance level. However, the results showed no alterations in Lee’s index, FBG or FINS in the HFD group compared to the HFDS group. We believe that the main reason for this phenomenon is the small size of the statistical sample. Thus, we will increase the size of the statistical sample to further examine the impact of Kaili Sour Soup on NAFLD in future studies. Furthermore, the results showed that there were no alterations in the indicators when comparing the NDS and ND groups, indicating that Kaili Sour Soup might exert effects only on the pathological processes of NAFLD. However, further research, including an epidemiological survey with a large sample size, is needed to determine the impacts of Kaili Sour Soup on NAFLD.

Recently, due to the advantages of high-throughput methods, such as genomics, proteomics and lipidomics, systematic analyses of genes, proteins, and metabolites have been utilized to provide more comprehensive information to understand the physiological and pathological processes of various diseases. Several studies have applied proteomic and lipidomic analyses to comprehensively dissect the homeostasis of proteins and lipids in NAFLD [9, 10]. However, no studies have focused on global changes in proteins and lipids in NAFLD treated with Kaili Sour Soup. The crucial proteins and lipids that are changed by Kaili Sour Soup, as well as their corresponding biological systems, can be identified via global profiling technologies, such as proteomics and lipidomics, thus greatly improving our understanding of the effects of Kaili Sour Soup on NAFLD. Therefore, we examined the protein and lipid profiles in the livers of NAFLD rats with and without treatment with Kaili Sour Soup via integrated proteomics and lipidomics analysis. Then, the deregulated proteins and lipids were used for integration analysis and network correlation and to identify signalling pathways, which are proposed to underlie the mechanism of the impact of Kaili Sour Soup in NAFLD.

Proteomics analysis revealed that there were 102 differentially expressed proteins (FC >  ± 1.2 and *p* < 0.05), of which 53 were increased and 49 were decreased in the HFD group compared to the ND group. Meanwhile, there were 27 differentially expressed proteins (FC >  ± 1.2 and *p* < 0.05), of which 6 were increased and 21 were decreased in the HFDS group compared to the HFD group. Among these differentially expressed proteins, 12 proteins were upregulated in the HFD group compared to the ND group and downregulated in the HFDS group compared to the HFD group, including Tmem44, Rnaseh2b, Gstm6l, LOC100910877, Rufy4, Slc12a2, Pcif1, P4503A1, Sult1e1, Nop53, AABR07065656.4, and AABR07065789.3. Furthermore, 3 proteins were downregulated in the HFD group compared to the ND group and upregulated in the HFDS groups compared to the HFD group, including Sult1c2, Sult1c2a, and Snrnp48. These proteins are critical proteins that might be implicated in the effect of Kaili Sour Soup on NAFLD.

Sulfotransferase family 1E (Sult1e1) encodes sulfotransferase E1, which plays a role in the inactivation of oestrogen [11]. The role of Sult1e1 in NAFLD is controversial. Chunhua Wang and colleagues showed that Sult1e1 was downregulated in NAFLD, and another study revealed that Sult1e1 was upregulated in nonalcoholic steatohepatitis [12, 13]. Our study showed that Sult1e1 was increased in the HFD group and decreased in the HFDS group, indicating that Sult1e1 might exert an important impact on HFD treatment with or without Kaili Sour Soup administration. Slc12a2 is also known as the Na+- K+-2Cl–cotransporter (NKCC1). The study by Alshahrani & Di Fulvio found that Slc12a2 knockout mice did not show a hyperglycaemia/diabetes phenotype [14]. Another study showed that mice lacking a single Slc12a2 allele exhibited lower fasting blood glucose, increased acute insulin response (AIR) and lower blood glucose levels 15–30 min after glucose loading [15]. The improvement of islet resistance is an important indicator of NAFLD remission. Pcif1 (also known as spop) is an inhibitor of PDX1 (the homeodomain transcription factor pancreatic duodenal homeobox 1). PDX1 is the main mediator of insulin transcription and the key regulator of the β-cell phenotype. Studies have shown that reducing the dose of the pcif1 gene can improve glucose tolerance and normalize the quality of β-cells to improve islet function and the development of NAFLD [16]. Cytochrome P450 is a type of ferriheme-containing superfamily protease that mainly includes three gene families: CYP1, CYP2 and CYP3 and is the main member of the biotransformation enzyme system and is involved in endogenous compounds (hormones, fatty acids) and exogenous compounds (drugs, precarcinogens). The enzyme activity, protein expression and mRNA expression levels of P450 3A1 in the livers of diabetic and obese rats and mice were higher than those of the normal group [17, 18]. Thus, further research is needed to elucidate the role played by Sult1e1, Slc12a2, Pcif1 and P450 3A1 in HFD treatment with or without Kaili Sour Soup and its underlying mechanism.

Global lipidomic analysis has been widely applied to identify biomarkers of NAFLD progression [19–21]. Here, we revealed that Kaili Sour Soup inhibited the HFD-induced elevation of AcCa and reversed the HFD-induced decreases in GM3 and LPS, indicating that these three lipid classes, AcCa, GM3, and LPS, might affect the role of Kaili Sour Soup in NAFLD.

As a component of fatty acyl compounds, AcCa is derived from fatty acid oxidation, and its levels measurably change in response to a variety of physiological factors [22, 23]. This study showed that the AcCa level was elevated in the HFD group, consistent with previous studies [24, 25]. However, no study has focused on the AcCa level after treatment with Kaili Sour Soup. Thus, it is worth further investigating the role played by AcCa in NAFLD with or without Kaili Sour Soup treatment and its underlying mechanism.

As the main ganglioside of the human liver, GM3 (sialosyl-lactosylceramide) accounts for more than 90% of the total lipid-bound sialic acid [26]. Robin B. Chan and colleagues discovered that the plasma concentration of GM3 was elevated in idiopathic Parkinson's disease [27]. To date, few studies have reported the levels of GM3 in NAFLD. Thomas N. Seyfried and colleagues revealed that the total amount of GM3 consisted of unsubstituted fatty acids (56.9%) and hydroxy fatty acids (43.1%), indicating a major role for GM3 in the metabolic balance of the liver [28]. This study showed that Kaili Sour Soup inhibited the HFD-induced increase in GM3 levels, indicating that GM3 plays a role in NAFLD treated with Kaili Sour Soup.

As a member of the phosphatidylserine (PS) family that mediates apoptotic cell clearance, LPS (lysophosphatidylserine) is a bioactive signalling phospholipid [29]. However, few studies have examined LPS levels in NAFLD. Here, we suggested that Kaili Sour Soup inhibited the HFD-induced increase in LPS levels, indicating that LPS plays a role in NAFLD treated with Kaili Sour Soup.

Increasing evidence has revealed that sex-related differences affect the prevalence, severity and major risk factors for NAFLD. For example, a higher incidence of hepatic tumours, more proinflammatory/profibrotic cytokines and severe steatosis and steatohepatitis were observed in animal models of NAFLD in males than in females, which was also observed in patients. In general, the prevalence and severity of NAFLD are higher in men than in women during reproductive age, and after menopause, NAFLD occurs at a higher rate in women, suggesting that oestrogen is protective [30–32]. These findings suggested that oestrogen is protective in the physiological and pathological process of NAFLD. However, this research did not systematically study the effect of gender on the function of Kaili Sour Soup in NAFLD thus affecting our conclusion: Kaili Sour Soup has a protective effect on NAFLD and affects liver protein and lipid metabolism in NAFLD. Moreover, Kaili Sour Soup had no protective effect on the female NAFLD animal model or corresponding female patients with NAFLD.

This is the first study to systematically examine the effect of Kaili Sour Soup on liver protein and lipid metabolism in NAFLD. To focus on the function of Kaili Sour Soup, we first excluded the variable of hormone function in NAFLD and confirmed the role and further investigated the underlying related molecular mechanism of Kaili Sour Soup in NAFLD; second, after confirming the protective function of Kaili Sour Soup on NAFLD, we will also study the effects of Kaili Sour Soup on liver transcriptome and intestinal flora of the NAFLD animal model. Therefore, after completing these works, we will introduce gender as a variable to study the effect of gender on the function of Kaili Sour Soup in NAFLD.

## Conclusion

This study revealed that Kaili Sour Soup exerted a protective effect in NAFLD by affecting the global proteome and lipidome, thus providing evidence for the beneficial impact of Kaili Sour Soup on a NAFLD rat model.

## Supplementary Information

**Additional file 1: Figure S1.** Proteins identified by TMT quantitative proteomics analysis.

**Additional file 2: Figure S2.** Lipid species identified by lipidomic analysis.

**Additional file 3: Figure S3.** Lipid classes identified by lipidomic analysis.

## Data Availability

The datasets used or analysed during the current study are available from the corresponding author on reasonable request.

## Abbreviations

NAFLD: Nonalcoholic fatty liver disease
HE: Haematoxylin and eosin
HFD: High-fat diet
TC: Total cholesterol
TG: Triglyceride
ALT: Alanine aminotransferase
AST: Aspartate aminotransferase
FBG: Fasting blood glucose
HOMA-IR: Insulin resistance level of homeostasis model assessment
AcCa: Acyl carnitine
GM3: Gangliosides
LPS: Lysophosphatidylserine
PS: Phosphatidylserine
NASH: Nonalcoholic steatohepatitis
LC–MS: Mass spectrometry
ND group: Normal standard feed + purified water gavage for 12 weeks
NDS group: Normal standard feed + Kaili Sour Soup gavage for 12 weeks
HFD group: High-fat feed + purified water gavage for 12 weeks
HFS group: High fat feed + Kaili Sour Soup gavage for 12 weeks
PFA: Paraformaldehyde
FASP: Filter aided sample preparation
TFA: Trifluoroacetic acid
AGC: Automatic gain control
RIPA: Radioimmunoprecipitation assay
BCA: Bicinchoninic acid
PVDF: Polyvinylidene fluoride
BSA: Bovine serum albumin
PCA: Principal component analysis
Sult1e1: Sulfotransferase family 1E
NKCC1: Na+- K+-2Cl–cotransporter
AIR: Acute insulin response
PDX1: Pancreatic duodenal homeobox 1

## References

[1] Xiao J, Guo R, Fung ML, Liong EC, Tipoe GL (2013). Therapeutic approaches to non-alcoholic fatty liver disease: past achievements and future challenges. Hepatobiliary Pancreat Dis Int.

[2] Sheth SG, Gordon FD, Chopra S (1997). Nonalcoholic steatohepatitis. Ann Intern Med.

[3] Neuman MG, Cohen LB, Nanau RM (2014). Biomarkers in nonalcoholic fatty liver disease. Can J Gastroenterol Hepatol.

[4] Smith R, Mathis AD, Ventura D, Prince JT (2014). Proteomics, lipidomics, metabolomics: a mass spectrometry tutorial from a computer scientist's point of view. BMC Bioinformatics.

[5] Higdon AN, Landar A, Barnes S, Darley-Usmar VM (2012). The electrophile responsive proteome: integrating proteomics and lipidomics with cellular function. Antioxid Redox Signal.

[6] Wisniewski JR, Zougman A, Nagaraj N, Mann M (2009). Universal sample preparation method for proteome analysis. Nat Methods.

[7] TerHorst KW, Serlie MJ (2017). Fructose consumption, lipogenesis, and non-alcoholic fatty liver disease. Nutrients.

[8] Ipsen DH, Lykkesfeldt J, Tveden-Nyborg P (2018). Molecular mechanisms of hepatic lipid accumulation in non-alcoholic fatty liver disease. Cell Mol Life Sci.

[9] Knebel B, Fahlbusch P, Dille M, Wahlers N, Hartwig S, Jacob S, Kettel U, Schiller M, Herebian D, Koellmer C (2019). Fatty liver due to increased de novo lipogenesis: alterations in the Hepatic Peroxisomal Proteome. Front Cell Dev Biol.

[10] Martel C, Esposti DD, Bouchet A, Brenner C, Lemoine A (2012). Non-alcoholic steatohepatitis: new insights from OMICS studies. Curr Pharm Biotechnol.

[11] Leiter EH, Chapman HD (1994). Obesity-induced diabetes (diabesity) in C57BL/KsJ mice produces aberrant trans-regulation of sex steroid sulfotransferase genes. J Clin Invest.

[12] Wang C, Tao Q, Wang X, Wang X, Zhang X (2016). Impact of high-fat diet on liver genes expression profiles in mice model of nonalcoholic fatty liver disease. Environ Toxicol Pharmacol.

[13] Matsushita N, Hassanein MT, Martinez-Clemente M, Lazaro R, French SW, Xie W, Lai K, Karin M, Tsukamoto H (2017). Gender difference in NASH susceptibility: roles of hepatocyte Ikkbeta and Sult1e1. PLoS ONE.

[14] Alshahrani S, Di Fulvio M (2012). Enhanced insulin secretion and improved glucose tolerance in mice with homozygous inactivation of the Na(+)K(+)2Cl(−) co-transporter 1. J Endocrinol.

[15] Alshahrani S, Almutairi MM, Kursan S, Dias-Junior E, Almiahuob MM, Aguilar-Bryan L, Di Fulvio M (2015). Increased Slc12a1 expression in beta-cells and improved glucose disposal in Slc12a2 heterozygous mice. J Endocrinol.

[16] Claiborn KC, Sachdeva MM, Cannon CE, Groff DN, Singer JD, Stoffers DA (2010). Pcif1 modulates Pdx1 protein stability and pancreatic beta cell function and survival in mice. J Clin Invest.

[17] Kudo T, Shimada T, Toda T, Igeta S, Suzuki W, Ikarashi N, Ochiai W, Ito K, Aburada M, Sugiyama K (2009). Altered expression of CYP in TSOD mice: a model of type 2 diabetes and obesity. Xenobiotica.

[18] Chen GM, Hu N, Liu L, Xie SS, Wang P, Li J, Xie L, Wang GJ, Liu XD (2011). Pharmacokinetics of verapamil in diabetic rats induced by combination of high-fat diet and streptozotocin injection. Xenobiotica.

[19] Gorden DL, Myers DS, Ivanova PT, Fahy E, Maurya MR, Gupta S, Min J, Spann NJ, McDonald JG, Kelly SL (2015). Biomarkers of NAFLD progression: a lipidomics approach to an epidemic. J Lipid Res.

[20] Svegliati-Baroni G, Pierantonelli I, Torquato P, Marinelli R, Ferreri C, Chatgilialoglu C, Bartolini D, Galli F (2019). Lipidomic biomarkers and mechanisms of lipotoxicity in non-alcoholic fatty liver disease. Free Radic Biol Med.

[21] Mann JP, Feldstein AE, Nobili V (2017). Update on lipid species and paediatric nonalcoholic fatty liver disease. Curr Opin Clin Nutr Metab Care.

[22] Schooneman MG, Vaz FM, Houten SM, Soeters MR (2013). Acylcarnitines: reflecting or inflicting insulin resistance?. Diabetes.

[23] Bray GA, Redman LM, de Jonge L, Rood J, Sutton EF, Smith SR (2018). Plasma fatty acyl-carnitines during 8weeks of overfeeding: relation to diet energy expenditure and body composition: the PROOF study. Metabolism.

[24] Xu C, Song D, Holck AL, Zhou Y, Liu R (2020). Identifying lipid metabolites influenced by oleic acid administration using high-performance liquid chromatography-mass spectrometry-based lipidomics. ACS Omega.

[25] Dahlhoff C, Worsch S, Sailer M, Hummel BA, Fiamoncini J, Uebel K, Obeid R, Scherling C, Geisel J, Bader BL, Daniel H (2014). Methyl-donor supplementation in obese mice prevents the progression of NAFLD, activates AMPK and decreases acyl-carnitine levels. Mol Metab.

[26] Riboni L, Acquotti D, Casellato R, Ghidoni R, Montagnolo G, Benevento A, Zecca L, Rubino F, Sonnino S (1992). Changes of the human liver GM3 ganglioside molecular species during aging. Eur J Biochem.

[27] Chan RB, Perotte AJ, Zhou B, Liong C, Shorr EJ, Marder KS, Kang UJ, Waters CH, Levy OA, Xu Y (2017). Elevated GM3 plasma concentration in idiopathic Parkinson's disease: a lipidomic analysis. PLoS ONE.

[28] Seyfried TN, Ando S, Yu RK (1978). Isolation and characterization of human liver hematoside. J Lipid Res.

[29] Frasch SC, Berry KZ, Fernandez-Boyanapalli R, Jin HS, Leslie C, Henson PM, Murphy RC, Bratton DL (2008). NADPH oxidase-dependent generation of lysophosphatidylserine enhances clearance of activated and dying neutrophils via G2A. J Biol Chem.

[30] Lonardo A, Nascimbeni F, Ballestri S, Fairweather D, Win S, Than TA, Abdelmalek MF, Suzuki A (2019). Sex Differences in Nonalcoholic Fatty Liver Disease: State of the Art and Identification of Research Gaps. Hepatology.

[31] Clayton JA, Collins FS (2014). Policy: NIH to balance sex in cell and animal studies. Nature.

[32] Miller VM, Rocca WA, Faubion SS (2015). Sex Differences Research, Precision Medicine, and the Future of Women's Health. J Womens Health (Larchmt).

