# Supplementary figures and images for: Integrative proteomic and lipidomic analysis of Kaili Sour Soup-mediated attenuation of high-fat diet-induced nonalcoholic fatty liver disease in a rat model
Source: Nutr Metab (Lond). 2021 Mar 10;18:26. doi: 10.1186/s12986-021-00553-4 (PMC7945315; doi:10.1186/s12986-021-00553-4)

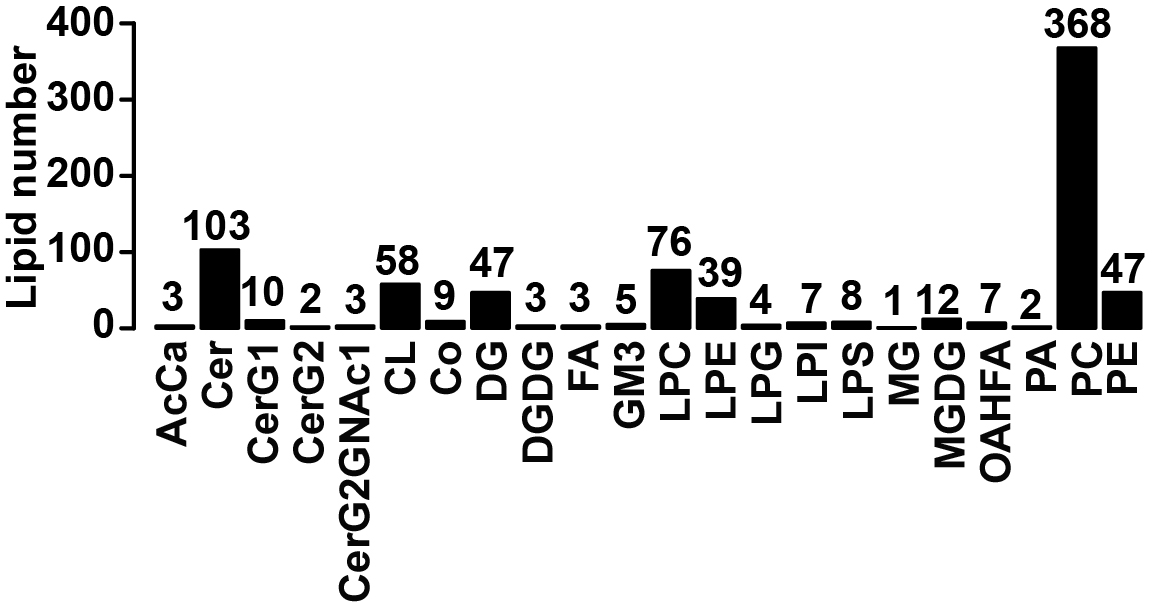

Supplement: Supplementary file 3 — Additional file 3: Figure S3. Lipid classes identified by lipidomic analysis. [file 12986_2021_553_MOESM3_ESM.jpg]
